# Sustainable exploitation of high-protein feather waste for green production of cold-adapted and detergent-stable keratinase by *Penicillium oxalicum* AUMC 15084

**DOI:** 10.1007/s11274-025-04417-4

**Published:** 2025-06-05

**Authors:** Ayat M. A. Abdel-Latif, Nageh F. Abo-Dahab, Ahmed M. Moharram, Abdallah M. A. Hassane, Osama A. M. Al-Bedak

**Affiliations:** 1 Department of Botany and Microbiology, Faculty of Science, Al-Azhar University, Assiut, 71524 Egypt; 2 Department of Botany and Microbiology, Faculty of Science, Assiut University, Assiut, 71511 Egypt; 3 Assiut University Mycological Centre, Assiut, 71511 Egypt; 4 ERU Science & Innovation Center of Excellence, Egyptian Russian University, Badr city, Cairo, 11829 Egypt

**Keywords:** Cold-adapted, Keratinase, *Penicillium*, Optimization, Purification

## Abstract

Limited research has investigated the ability of psychrophilic and psychro-tolerant microorganisms to produce cold-active keratinases, despite their potential as an efficient alternative for substrate conversion at reduced energy expenditure. A screening of 32 *Penicillium* and *Talaromyces* isolates for keratinolytic activity at temperatures of 5, 10, and 15ºC identified a promising *P. oxalicum* strain as the most potent at 10ºC, yielding 242.39 U/mL. Following six days of incubation at pH 8.0 and 15 °C with 0.2% yeast extract as the nitrogen source, the *P. oxalicum* strain exhibited keratinase activity of 359.42 U/mL. The keratinase underwent purification with a 4.13-fold increase, utilizing an MP 800 anion exchanger and Sephacryl S 200 , resulting in a specific activity of 684.46 U/mg and a yield of 5.34%. The SDS-PAGE analysis identified a keratinase with a molecular weight of 37.51 kD, exhibiting peak activity at pH 9.0 and 20ºC, with a specific activity of 721.8 U/mg. Mg^2+^, Zn^2+^, and Mn^2+^ enhanced keratinase activity by 156.0%, 140.60%, and 156.0%, respectively. The keratinase activity was significantly enhanced (*p* < 0.05) by the addition of 5 mM SDS (139.15%), 5 and 10% mercaptoethanol (1125.70 and 1327.0%, respectively), and 5 and 10% DMSO (128.30 and 227.40%, respectively). The dehairing potential of *P. oxalicum* AUMC 15084, utilizing crude keratinase on goat skin, demonstrated complete dehairing after 20 h at 20ºC with the crude preparation. This study provides a promising *Penicillium oxalicum* strain that could be used for production of cold-active keratinase. The effectiveness of the produced keratinase in the dehairing process was demonstrated as an environmentally friendly alternative to the traditional chemical procedure.

## Introduction

Continuous environmental rehabilitation, the growth of an environmentally conscious economy, and enhanced quality of life are all facilitated by environmentally friendly production and consumption (Al Mousa et al. 2021). Innovative renewable products have had significant popularity in recent decades, outpacing that of synthetic ones. In most economies trying to boost biotechnology through biodiversity, renewable resources are preferred over synthetic ones due to the opportunities they present. However, this discipline is still in its early stages because non-renewable resources account for the majority of the resources that are accessible (Nnolim et al. 2020). Microbial enzymes are extensively used in the bio-economic domain (Al Mousa et al. 2021). The global enzyme market, including the many types of enzymes utilized by various industries, was estimated to be worth 5.5 billion US$ in 2018 and was projected to grow to 7.0 billion US$ by 2025 (Bhatia et al. 2021). As a result, researchers are currently focused on discovering new enzymes that have the potential to replace industrial chemical catalysis (Al Mousa et al. 2021). Enzymes are progressively replacing chemical processes in industry due to their greater environmental friendliness, capacity to raise product value, decreased production of waste products, low energy requirements, and less environmental pollution (Sarmiento et al. 2015; Bruno et al. 2019). In the industrial manufacturing of commodities on a commercial scale, standard enzymes are often subjected to variations of pH, temperature, and salinity, which can cause denaturation (Sarmiento et al. 2015).

Wool, hooves, horns, hair, nails, and feathers all contain keratins as their structural elements (Qiu et al. 2020). The United States, Brazil, and China together produce 40 million tons of keratinous waste annually (Sharma and Gupta 2016; Alwakeel et al. 2021). Millions of tons of keratinous waste are produced worldwide each year by the meat-producing industry, namely at slaughterhouses (Verma et al. 2017). The chicken sector produces over 3.4 million tons of feather waste yearly, according to EU data (Reddy et al. 2021). Keratins are insoluble fibrillar proteins found on the external protective coverings of animals. Because their polypeptide chains are strongly stabilized and have several disulfide connections spanning them, keratinases are specifically hydrolyzing keratinous substrates, which include feathers, wool, and nails (Hassan et al. 2020a). Two of the numerous potential uses for microbial keratinases, which have recently gained popularity in biotechnology, are the bio-conversion of resistant keratin-rich wastes and the long-term adoption of cleaner production (Nnolim et al. 2020; Alwakeel et al. 2021; Al-Bedak et al. 2023).

Cold-adapted enzymes can still catalyze, even at 30 °C and at 0 °C, they can still retain some catalytic activity (Liu et al. 2023). Psychrophile excretion is a highly underutilized source of cold-adapted enzymes that exhibit high specific activity at low temperature. Differentiating them from mesophilic and thermophilic enzymes are their low reaction energy (the ideal catalytic temperature is usually between 20 and 45 °C), higher substrate affinity that can lower the activation energy of enzymatic reactions, and low thermal stability at high temperatures that cause them to quickly lose more than half of their activity after 10 min at 50–60 °C or several hours at 37 °C (Santiago et al. 2016; Moharram et al. 2022).

Mesophilic and thermophilic organisms are the sources of most keratinases that have been identified to date (Alwakeel et al. 2021; Sittipol et al. 2021; Al-Bedak et al. 2023; Derhab et al. 2023; Kokwe et al. 2023; Pei et al. 2023). While psychrophiles and psychro-tolerant microorganisms have yielded the majority of cold-active enzymes, mesophilic and even thermophilic species have also produced certain enzymes with significant low-temperature activity (Santiago et al. 2016). Although psychrophilic and psychrotolerant microorganisms can be a helpful alternative for converting substrates at a low energy cost, there haven’t been many investigations on their ability to produce cold-active keratinases (Joshi and Satyanarayana 2013). As a result, these enzymes have been extensively researched and used in a variety of fields, such as basic molecular biology research, food processing, detergent manufacturing, bioremediation, environmental protection, and straw resourcing (Yadav et al. 2022). Consequently, the purpose of this work is to produce cold-active keratinase from psychrophilic *Penicillium oxalicum* AUMC 15084 in a manner that is both economically and environmentally sustainable, as well as to purify the enzyme and use it for the purpose of dehairing animal skin.

## Materials and methods

### Microorganisms

Thirty isolates from five different *Penicillium* species and two *Talaromyces*-related strains were employed in this study. Every strain that had been tested was acquired from the culture collection of Assiut University Mycological Centre (AUMC), Assiut Governorate, Egypt. These were namely *Penicillium brevicompactum* (2 isolates), *P. chrysogenum* (12), *P. solitum* (8), *P. griseofulvum* (4), *P. oxalicum* (4), and *Talaromyces duclauxii* (2).

### Extraction of keratin

After being gathered from poultry farm in the Assiut Governorate of Egypt, 50 g of chicken feathers were defatted with a 1:1 mixture of chloroform and methanol for a full day while being continuously stirred. The feathers were then cleaned using distilled water and allowed to dry in the air. During six hours at room temperature and constant stirring, 1000 mL of 0.5 M sodium sulfide were submerged with the chicken feathers to extract keratin. 70% ammonium sulfate was used to extract soluble keratin from the supernatant after it had been centrifuged for 10 min at 10,000 ×g. Precipitate was utilized as chicken keratin powder in keratinase test assays after being dried at 40 °C and cleaned four times with distilled water (Alwakeel et al. 2021; Al-Bedak et al. 2023).

### Fermentation medium and fermentation conditions

This study used Czapek’s broth, which is free of sucrose, as a fermentation medium. The medium contained (g/L): Sodium nitrate, 2; potassium chloride, 0.5; magnesium sulphate, 0.5; zinc sulfate, 0.01; and copper sulphate, 0.005. One mL of the tested fungus’s cell suspension was added to a 250 mL Erlenmeyer flask containing 50 mL fermentation medium supplemented with 1.0% native chicken feathers as the only source of carbon. Cell suspension of each examined strain (1.5 × 10^8^ spores/mL) from 7-day-old cultures was used separately to inoculate the flasks. The flasks were then kept at 5, 10, and 15 °C under shacked conditions (150 rpm) for 10 days. After fermentation time, the cell-free supernatants were obtained by centrifugation (10,000 rpm for 10 min at 4 °C), and used as keratinase source in the assay experiment.

### Keratinase assay

One mL of the fungal supernatant was reacted with 0.01 g of the pure keratin (dissolved in 1.0 mL of phosphate buffer, pH 8.0). The reaction was stopped after 60 min at 10 °C by introducing 2.0 mL of 10% Trichloroacetic acid (TCA). The reaction’s contents were then centrifuged (10,000 rpm for 10 min at 4 °C), and 5.0 mL of the alkaline copper reagent (sodium carbonate, 40 g; tartaric acid, 7.5 g; copper sulfate, 4.5 g; and distilled water, 1000 mL; pH 10 ± 0.5) were added after 0.2 mL of the supernatant had been diluted to 1.0 mL. After that, 0.5 mL of the Folin-Ciocalteau reagent was added and the tubes were kept 30 min in darkness to allow the development of the blue color. Absorbance was then measured at 660 nm (UV-visible spectrophotometer; T80+; Leicestershire, UK), and the keratinase activity was calculated employing Tyrosine as standard. One unit (U) of keratinase activity is defined as the amount of the enzyme required to release one µmol of tyrosine per minute.

### Morphological and molecular identification of the potent strain

Using an inoculum size of 1 µL/spot, plates were cultured in a three-point pattern on Czapek’s agar (Cz), malt extract agar (MEA), and Czapek’s Yeast Autolysate agar (CYA) (Smith and Onions 1994), using spore suspension, a mixture of 30% glycerol, 0.2% agar, and 2 drops of Tween 80 with a Zeiss microscope (Model: Axio Star; Jena, Germany), the microscopic features of CYA were assessed with lactophenol cotton blue following a 7-day incubation period at 25 °C. For molecular confirmation of the potent strain, DNA of the fungus in this study was isolated (Moubasher et al. 2019), and the PCR reaction was performed at SolGent Co. (South Korea), using SolGent EF-Taq (Al-Bedak and Moubasher 2020). For ITS region amplification, ITS1 and ITS4 universal primers were used (White et al. 1990). Contiguous sequence of the *Penicillium oxalicum* included in this study was produced using the DNASTAR (version 5.05). The total ITS dataset for phylogenetic analysis contained 17 sequences, sequence of *Talaromyces pinophilus* CBS 631.66 as outgroup, sequence for *Penicillium oxalicum* AUMC 15084 in this work, and 15 sequences retrieved from GenBank related to the genus *Penicillium*. All sequences were aligned together using MAFFT (version 6.861b) with the default options (Katoh and Standley 2013). BMGE (Criscuolo and Gribaldo 2010) was used to optimize the alignment gaps and parsimony uninformative characters. Maximum-likelihood (ML) and maximum-parsimony (MP) phylogenetic analyses was performed by MEGA X (version 10.2.6) (Kumar et al. 2018), and the robustness of the most parsimonious trees was applying 1000 replications (Felsenstein 1985). Utilizing Modeltest 3.7’s Akaike Information Criterion (AIC), the optimum nucleotide substitution model for ML analysis was identified (Posada and Crandall 1998). The resulting tree was edited and saved as TIF format (Al-Bedak et al. 2020).

### Improvement of keratinase production

By applying the two factors at a time (TFAT) and adjusting the pH (3, 4, 5, 6, 7, 8, 9, and 10) at 10, 15, and 20 °C, the culture conditions were optimized. Fermentation times ranging from one to seven days were optimized for the nitrogen supply (peptone, yeast extract, beef extract, sodium nitrate, ammonium chloride, and ammonium sulphate; each at 0.2%) at the ideal pH and temperature (Moharram et al. 2022; Al-Bedak et al. 2023). Fifty mL of the fermentation medium supplemented with 1% native chicken feathers were added to each of the 250 mL Erlenmeyer flasks. Each flask was then individually inoculated with a 5% spore suspension (1.5 × 10^8^ spore/mL) that was obtained from fungal cultures that had been cultivating for seven days. The citrate buffer (pH 3–6), phosphate buffer (pH 7–8), and citrate buffer (pH 3-6) were the buffers utilized for pH correction.

### Amino acid analysis of the crude keratinase

The amino acid analysis and determination were carried out by the Chromatography Laboratory at the National Research Centre, Giza, Egypt, using the techniques that were outlined by Campanella et al. (2002), Laurens et al. (2012), and Jajić et al. (2013). A mixture of 0.1 g of pure keratinase, 5.0 mL of water, and 5.0 mL of 6 M HCl was combined, and it was heated to 120 °C for 24 h before filtering. After being dried and reconstituted in 0.1 M HCl, a 1.0 mL of the filtrate was finally put to an Agilent 1260 series HPLC. For the separation, Eclipse Plus C18 column (4.6 mm × 250 mm; 5 μm diameter) was employed. A solvent mixture of acetone, methanol, and water (45: 45: 10) was combined with sodium phosphate dibasic/sodium borate buffer (pH 8.2) at a flow rate of 1.5 mL/min to generate the mobile phase. The mobile phase was programmed in sequential order using a linear gradient.

### Production of keratinase and precipitation by absolute Ethyl alcohol

Centrifugation was used to extract the culture supernatant once the enzyme reached its peak output under optimal growth conditions (10,000 rpm for 15 min at 4 °C). The clear supernatant was being gently stirred at 4 °C when cold absolute ethyl alcohol (-25 °C) was gradually added. The precipitated protein was then isolated, lyophilized, and used in the purification process.

### Purification of the cold-active keratinase

#### Lewatit MonoPlus (MP 800) anion exchange column

Pre-activated MP 800 gel with a bed capacity of 200 cm^3^ was placed inside a glass column measuring 60 cm × 2.4 cm. Ten milliliters of the crude keratinase sample were loaded to the ion exchange gel. The bound proteins were extracted using phosphate buffer (100 mmol, pH 9.0) and gradient NaCl concentrations ranging from 0 to 1.5 M, at a flow rate of 0.5 mL/min. Measurements of protein content and keratinase activity were made in fractions of 6.0 mL that were collected. In subsequent purification stages, the most active fractions were combined, concentrated, and used in further purification steps.

#### Sephacryl S 200 h gel filtration column

A Sephacryl S 200  gel filtration column (80 cm × 2.4 cm) was used to further separate the concentrated keratinase sample after it had buffered with phosphate buffer (100 mM, pH 9.0). Utilizing the same buffer, the protein was eluted at a 15 mL per hour flow rate. The most active fractions were combined, lyophilized, and used in the characterization tests.

#### Impact of pH and temperature on the activity of pure keratinase

First, 0.01 g of the purified enzyme was reacted at 15 °C with 0.01 g pure keratin (each dissolved in 1.0 mL buffer solution; pH 5–10), to determine the optimum pH of keratinase. Using the optimum pH for the enzyme, activity of the purified keratinase was estimated at 5–30 °C.

#### Impact of organic solvents and metal ions on the activity of pure keratinase

At the optimum pH and temperature, the effect of organic solvents such as methanol, ethanol, acetone, benzene, chloroform, hexane, toluene, and dimethyl sulfoxide (DMSO) each at concentrations of 5% and 10%, was estimated. Monovalent and divalent metal ions (Na^+^, K^+^, Ca^2+^, Co^2+^, Ni^2+^, Cu^2+^, Fe^2+^, Mn^2+^, Mg^2+^, and Zn^2+^) were evaluated by introducing them at 5 mmol/mL concentrations as NaCl, KCl, CaCl_2_, CoCl_2_, NiCl_2_, CuSO_4_, FeSO_4_, MnSO_4_, MgSO_4_, and ZnSO_4_ (Al-Bedak et al. 2023). Phenylmethylsulfonyl fluoride (PMSF), ethylenediaminetetraacetic acid (EDTA), and sodium dodecyl sulphate (SDS) were investigated at 5.0 mM. 2-mercaptoethanol and dimethylsulfoxide (DMSO) were tested at concentrations of 5.0 and 10.0%. In the absence of the tested chemical, the enzyme activity was regarded as 100%.

#### Determination of K_*m*_ and V_*max*_

By conducting a pure keratin experiment at various substrate concentrations ranging from 10 to 100 mg/mL, the kinetic constants for the keratinase activity, K_*m*_ and V_*max*_, were ascertained according to Lineweaver and Burk (1934).

#### Determination of keratinase molecular weight by SDS-PAGE

A 0.1 g of the pure keratinase was suspended in 100 µL of 20 mM Tris/HCl, pH 7.4 (Invitrogen, USA), which contained 2.5% bromophenol blue (tracking dye), 4.0% sodium dodecyl sulfate (SDS), 20% glycerol, and 10% of 2-mercaptoethanol. 12% SDS polyacrylamide gel was used for running at 100 mA and 150 V for 45 min and cooked for five minutes at 100 °C. For protein isolation, Coomassie Brilliant blue dye (R-250) was utilized. Gel was immersed in a de-staining solution after unbound dye was removed and blue bands representing the stained proteins were visible. The gel was then taken a picture with Quantity One program (Version 4.6.2).

### Dehairing activity

The ability of *Penicillium oxalicum* AUMC 15084 crude keratinase to remove epidermal hairs was studied using goat skin that was taken from a nearby slaughterhouse in Assiut Governorate, Egypt. The goat skin was repeatedly cleansed with tap and distilled water to remove the dirt and blood. Skin strips measuring 5 cm × 10 cm were removed after washing and left to air dry. Following the method described by Rehman et al. (2017), the culture filtrate of *P. oxalicum* AUMC 15084 strain was employed, with distilled water serving as the control. The goat skin was allowed to air dry before being dipped in 50 mL of crude enzyme and allowed to rest at 20ºC. After being incubated, the goat skin was removed from the supernatant and gently cleaned with distilled water.

### Statistical analysis

All data were expressed using the mean and standard deviation (SD) of the preliminary investigation, which was carried out in triplicate. Stahle and Wold (1989) method of statistical significance analysis was used. At *p* ≤ 0.05, it was considered significant.

## Results

### Screening of keratinase activity by *Penicillium* and *Talaromyces* species

Thirty isolates of *Penicillium* and two isolates of *Talaromyces duclauxii* were tested for their capacity to produce keratinase in SmF. For isolates that produced a lot of protein, submerged fermentation at 10 °C was utilized to perform the quantitative keratinase test using the sucrose-free CZ broth medium. The activity of the keratinase enzyme was measured at 5, 10, and 15 °C. The isolates’ production ranged from 5.11 U/mL to 242.39 U/mL of keratinase. *Penicillium griseofulvum* AUMC 309 and *Penicillium brevicompactum* AUMC 528 were the only two isolates identified to be powerful producers at 5 °C, with 206.45 U/mL and 204.41 U/mL, respectively. Eight were weak, while twenty-two were moderate. At 10 °C, twenty produced poorly, four in the moderate, and eight exceptionally well. At 15 °C, *Penicillium chrysogenum* AUMC 13364 was the only sample to produce 207.48 U/mL, while five were moderate and the other 26 isolates produced at low levels. The keratinase enzyme in *Penicillium* species is most active at 10 °C (Table 1). The most active strain at all was *Penicillium oxalicum* AUMC 15084 producing 242.39 U/mL, followed by *Penicillium solitum* AUMC 15155 (238.52 U/mL), *Talaromyces duclauxii* AUMC 14746 (235.23 U/mL), and *Penicillium chrysogenum* AUMC 2050 (233.67 U/mL).

**Table 1:** Keratinolytic activity of thirty-two strains of *Penicillium* and *Talaromyces* at 5, 10, and 15 °C in SmF

Penicillium species	Isolates count	Positive isolates	At 5 °C			At 10 °C			At 15 °C		
			H	M	W	H	M	W	H	M	W
*Penicillium brevicompactum*	2	2	1	1				2			2
*P. chrysogenum*	12	12		8	4	3	3	6	1		11
*P. solitum*	8	8		5	3	4	1	3		3	5
*P. griseofulvum*	4	4	1	3				4			4
*P. oxalicum*	4	4		3	1			4		2	2
*Talaromyces duclauxii*	2	2		2		1		1			2
**Total**	**32**	**32**	**2**	**22**	**8**	**8**	**4**	**20**	**1**	**5**	**26**

H = high producers: > 200 U/mL; M = moderate: > 100–200 U/mL; W = weak: < 100 U/mL

### Morphological and molecular identification of the potent strain

**Fig. 1:**
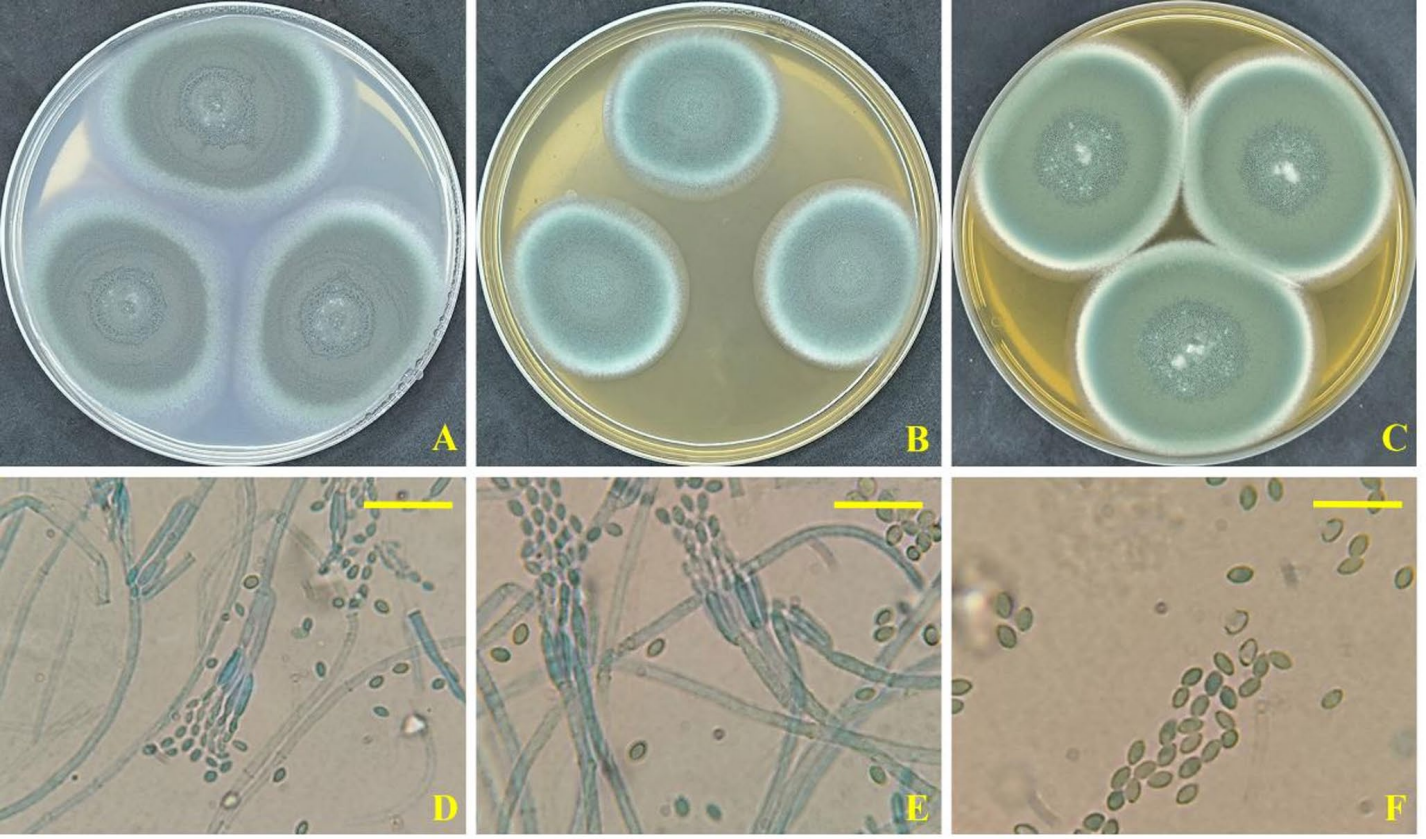
*P. oxalicum* AUMC 15084. A-C, Seven-day-old colonies on Cz, MEA, and CYA at 25 °C. D-E, conidiophores and penicilli. F, smooth, elliptical conidia (Scale bars = 20 μm)

**Fig. 2:**
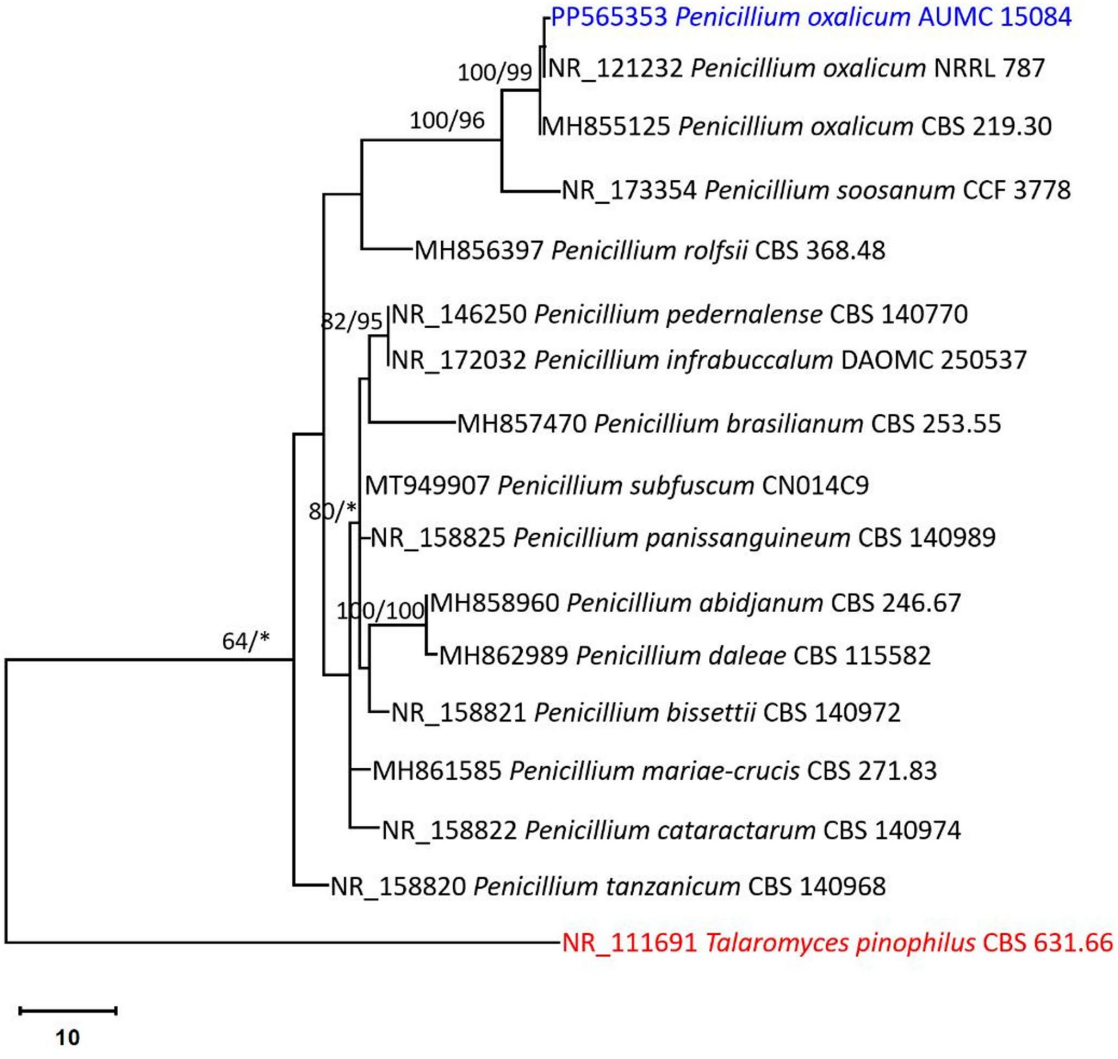
Maximum parsimony phylogenetic tree produced by ML/MP analysis using *P. oxalicum* AUMC 15084 ITS sequence data (in blue) in comparison to the most comparable *Penicillium* ITS sequences found in GenBank. Near the corresponding nodes, bootstrap support values (1000 replications) for ML/MP ≥ 50% are displayed. Tree is rooted to *Talaromyces pinophilus* CBS 631.66 (in red)

The strain used in this investigation displayed identical morphological characteristics as the type species *Penicillium oxalicum*. Colonies with rapid growth, sporulation heavy, grey-green. Conidiophores usually biverticillate, smooth-walled, up to 200 µm × 3.5–4.5 µm. Metulae 20–26 × 2.5–3.3 µm. Phialides 12.5–16 × 2.5–3.3 µm with a short, tapering neck. Conidia elliptical, smooth-walled, 4.2-6.0 × 2–3 µm (Fig. 1). Utilizing *P. oxalicum* AUMC 15084’ ITS sequence, the GenBank database revealed that *P. oxalicum* CBS 219.30 [(GenBank accession number MH855125; identities = 573/574 (99.83%); Gaps = 1/574 (0%)] was the most similar species. The strain in this study was positioned in the evolutionary tree at the *P. oxalicum* clade on the same branch as the type strain *P. oxalicum* NRRL 787, and *P. oxalicum* CBS 219.30, endorsing strong support clade with 100% ML/99% MP (Fig. 2). Thus, *P. oxalicum* was verified as the strain’s identity. The ITS sequence of *P. oxalicum* AUMC 15084 used in this investigation was registered as PP565353 in GenBank (Fig. 2).

### Effect of medium’s pH and incubation temperature on keratinase production

**Fig. 3:**
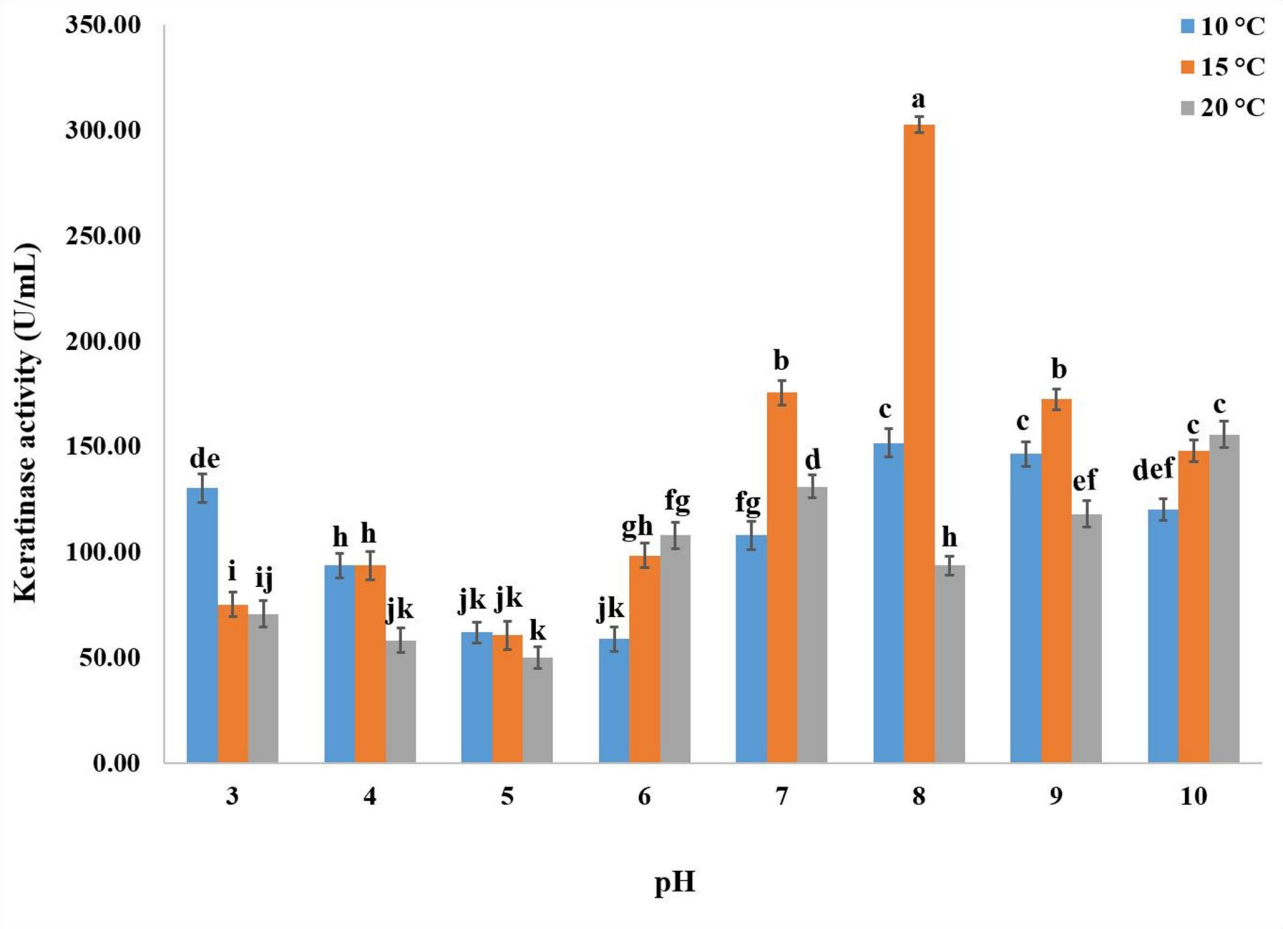
Effect of medium’s pH and incubation temperature on the keratinase production by *P. oxalicum* AUMC 15084 in SmF (Mean ± SD with different letters are significantly different (*p* < 0.05; *n* = 3)

**Fig. 4:**
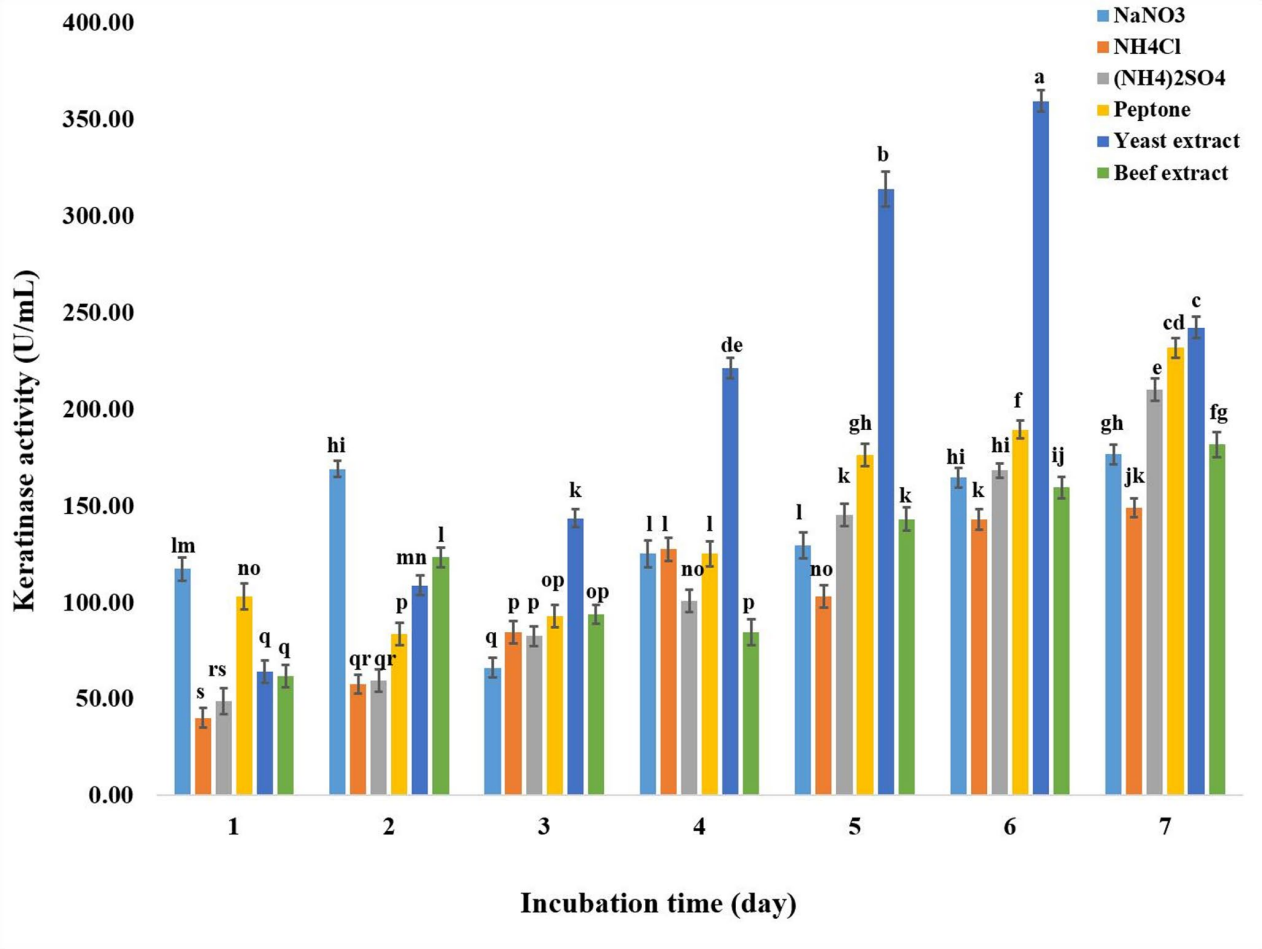
Effect of medium’s nitrogen source addition and incubation time on the keratinase production by *P. oxalicum* AUMC 15084 in SmF (Mean ± SD with different letters are significantly different (*p* < 0.05; *n* = 3)

According to the current data, keratinase production was considerably influenced by pH 8 and 15 °C, with a value of 302.53 ± 3.8 U/mL (Fig. 3). After six days of fermentation at pH 8 and 15 °C, the addition of 0.2% yeast extract significantly increased the synthesis of keratinase to 359.42 ± 5.4 U/mL in comparison to other nitrogen sources utilized (Fig. 4).

### Amino acids analysis

**Fig. 5:**
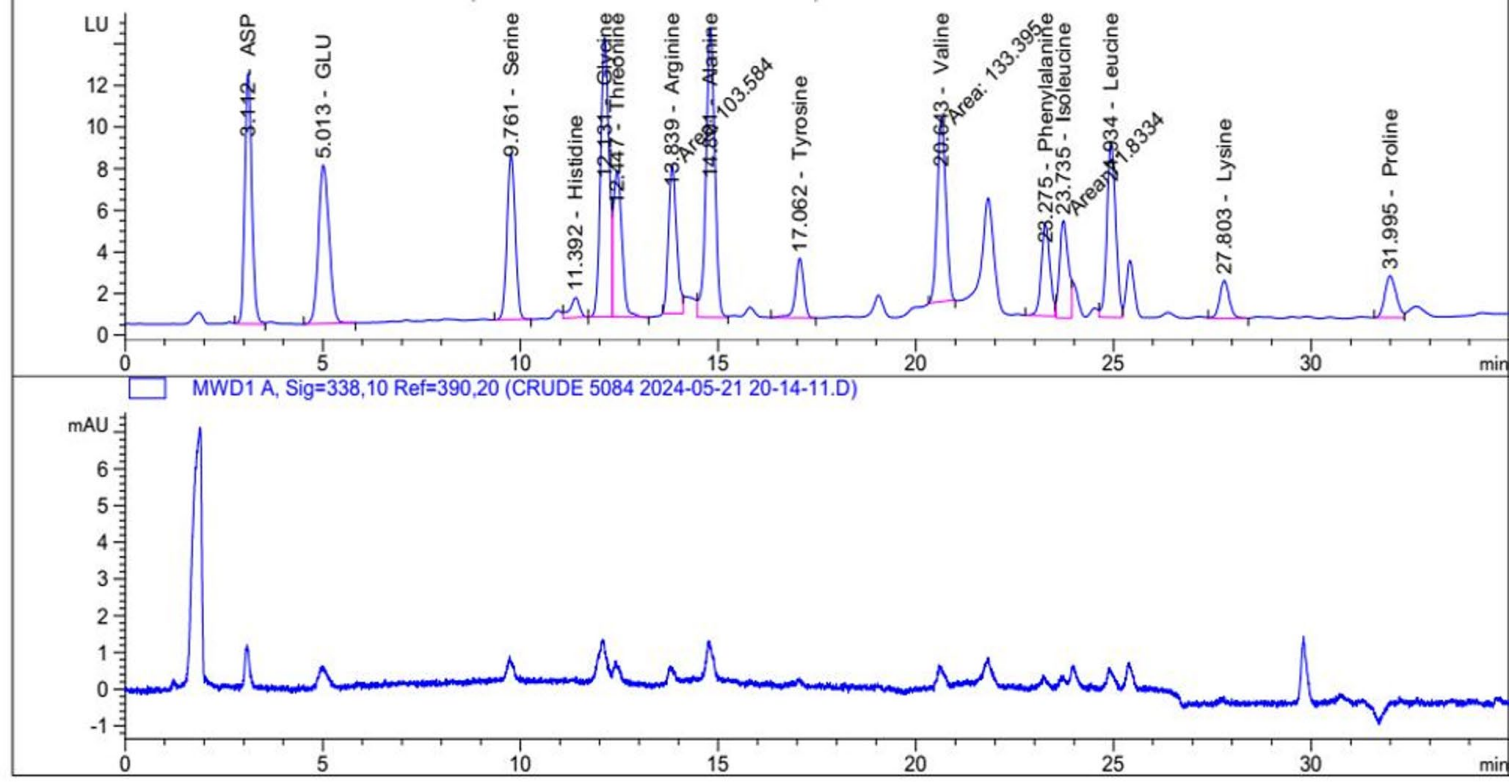
Amino acid profile of the crude cold-active keratinase produced by *P. oxalicum* AUMC 15084

This study looked into the composition of free amino acids in *P. oxalicum* AUMC 15084’s feather hydrolysate. The isolated *P. oxalicum* AUMC 15084’s keratinase included significant concentrations of aspartate (4.8 mg/g), glutamic acid (5.42), and lysine (5.56). Moderate levels were found in arginine (3.13 mg/g), lycine (3.09), and alanine (3.81). Less than 4 mg/g are made up of leucine (2.78 mg/g), serine (2.65), and threonine (2.58) respectively. Isoleucine, histidine, phenylalanine, and valine made up around (2.15, 1.82, 1.55, 0.97 mg/g). Tyrosine was 1.48 mg/g, while proline was 2.17 mg/g (Fig. 5).

### Purification profile of the cold-active keratinase

In the ideal submerged fermentation environment, *P. oxalicum* AUMC 15084 was able to excrete keratinase which was then purified using MP 800 anion exchanger and Sephacryl S 200 h. The pure keratinase activity was purified by 4.13-fold displaying 684.46 U/mg with 5.34% yield (Table 2).

**Table 2:** Purification profile of the cold-active keratinase produced by *P. oxalicum* AUMC 15084 at pH 8.0 and 15 °C after 6 days using yeast extract as nitrogen supply in SmF

Purification steps	Volume (mL)	Activity (U/mL)	Total activity (U)	Protein (mg/mL)	Total protein (mg)	Specific Activity (U/mg)	Yield (%)	Fold
Fermentation medium	1580	158.0	249,640	0.953	1505.74	165.8	100	1
Ethyl alcohol (absolute)	110	252.6	27,786	4.24	466.4	59.6	11.13	0.36
MP 800	65	330.6	21,489	1.7	110.5	194.47	8.6	1.17
Sephacryl S-200	30	444.9	13,347	0.65	19.5	684.46	5.34	4.13

### Molecular mass determination by SDS-PAGE

**Fig. 6:**
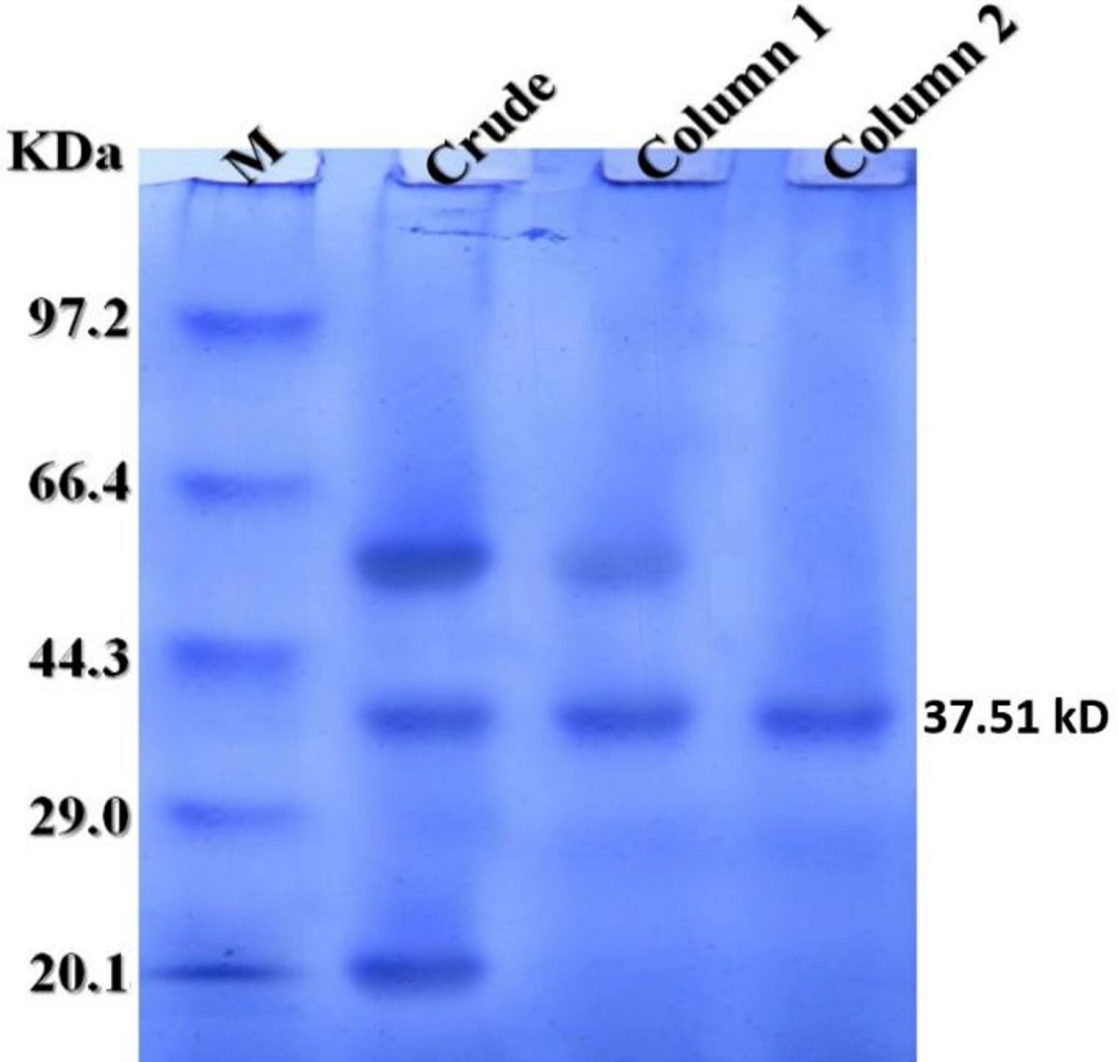
SDS-PAGE of the cold-active keratinase produced by *P. oxalicum* AUMC 15084 showing: Lane 1 (M) standard marker; Lane 2: crude keratinase; Lane 3: MP 800 column; and Lane 4: Sephacryl S 200

Results revealed that the pure cold-active keratinase in this study displayed a molecular weight of 37.51 kD (Fig. 6).

### Effect of pH and temperature on the activity of pure cold-active keratinase

**Fig. 7:**
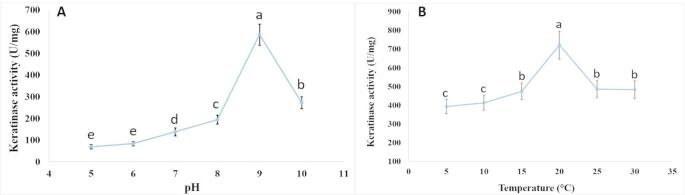
**(A)** Effect of pH on the activity of pure keratinase and **(B)** Effect of temperature on the activity of pure keratinase produced by *P. oxalicum* AUMC 15084 (Mean ± SD with different letters are significantly different (*p* < 0.05; *n* = 3)

When keratinase activity was measured between pH 4 and 10, the results showed that at pH 9.0, it reached 586 ± 48.6 U/mg (Fig. 7A). The enzyme activity rose to 721.8 ± 75 U/mg at 20 °C (Fig. 7B).

### Impact of metal ions and organic solvents on the activity of pure keratinase

The addition of various ions has varying impacts on keratinase activity. The only metal ions that significantly (*p* < 0.05) raised enzyme activity were Mg^2+^, Zn^2+^, and Mn^2+^; their percentages against the control were, respectively, 156.0 ± 2.9, 140.6 ± 1.75, and 156.0 ± 2.9%. The activity of the enzyme was not significantly affected by Na^+^, K^+^, Ca^2+^, Co^2+^, Cu^2+^, Ni^2+^, and Fe^2+^ all reduced the enzyme activity, while the largest rate of reduction was seen at 5.0 mM for Cu^2+^ (Table 3).

**Table 3:** Effect of metal ions on the activity of the pure keratinase produced by *P. oxalicum* AUMC 15084 (Mean values ± sd with different letters are significantly different (*p* < 0.05; *n* = 3)

Additions	Specific activity (U/mg)	Residual activity (%)
Control	721.8 ± 15.26^c^	100 ± 4.67^c^
NaCl	721.8 ± 25.55^c^	100 ± 3.54^c^
KCl	595.8 ± 20.93^d^	82.55 ± 2.9^d^
CaCl_2_	268.0 ± 29.16^c^	98.6 ± 4.0^c^
MgSO_4_	1126.9 ± 20.93^a^	156.0 ± 2.9^a^
FeSO_4_	292.8 ± 33.71^f^	40.6 ± 4.67^f^
CuSO_4_	78.3 ± 29.16^g^	10.9 ± 4.0^g^
ZnSO_4_	1014.6 ± 12.63^b^	140.6 ± 1.75^b^
MnSO_4_	1126.9 ± 20.93^a^	156.0 ± 2.9^a^
CoCl_2_	323.4 ± 20.93^f^	44.8 ± 2.9^f^
NiCl_2_	609.2 ± 20.93^d^	84.4 ± 2.9^d^

Data were presented as the mean of three replicates (Mean ± SD). Values followed by the different letters are significantly different at *p* < 0.05

### Impact of reducing agents, detergents, and inhibitors on keratinase activity

The keratinase activity was significantly enhanced (*p* < 0.05) by the addition of 5 mM SDS (139.15 ± 8.4%), 5 and 10% mercaptoethanol (1125.7 ± 69.4 and 1327.0 ± 113.57%, respectively), and 5 and 10% DMSO (128.3 ± 4.67 and 227.4 ± 29.9%, respectively). All the organic solvents and inhibitors used decreased the keratinase activity (Table 4).

**Table 4:** Effect of metal ions, inhibitors, organic solvents and reducing agents on the activity of the pure keratinase produced by *P. oxalicum* AUMC 15084 (Mean values ± sd with different letters are significantly different (*p* < 0.05; *n* = 3)

Additions	Specific activity (U/mg)	Residual activity (%)
Control	721.8 ± 15.26^def^	100 ± 4.67^def^
EDTA (5 mM)	718.2 ± 29.16^def^	99.5 ± 4.0^def^
SDS (5 mM)	1004.4 ± 60.93^f^	139.15 ± 8.4^f^
PMSF (5mM)	486.9 ± 30.93^abcd^	67.5 ± 4.3^abcd^
5% Hexane	616.3 ± 40.9^cde^	85.4 ± 5.67^cde^
10% Hexane	595.9 ± 29.16^bcde^	82.6 ± 4.0^bcde^
5% Toulene	534.6 ± 25.26^bcd^	74.0 ± 3.5^bcd^
10% Toulene	527.8 ± 33.7^bcd^	73.0 ± 4.67^bcd^
5% Chloroform	711.6 ± 40^def^	98.6 ± 5.5^def^
10% Chloroform	432.4 ± 20.9^abcd^	59.9 ± 2.9^abcd^
5% Benzene	544.7 ± 29.16^bcd^	75.5 ± 4^bcd^
10% Benzene	415.4 ± 33.7^abcd^	57.5 ± 4.67^abcd^
5% Methanol	211.0 ± 33.7^ab^	29.2 ± 4.67^ab^
10% Methanol	211.0 ± 20.93^ab^	29.2 ± 2.9^ab^
5% Ethanol	415.4 ± 25.26^abcd^	57.6 ± 3.5^abcd^
10% Ethanol	330.3 ± 20.93^abcd^	45.8 ± 2.9^abcd^
5% Acetone	313.2 ± 16.7^abc^	43.4 ± 2.3^abc^
10% Acetone	238.34 ± 15.9^abc^	33.0 ± 2.2^abc^
5% DMSO	926.0 ± 33.7^ef^	128.3 ± 4.67^ef^
10% DMSO	1641.0 ± 215.7^g^	227.4 ± 29.9^g^
5% 2-Mercaptoethanol	8125.3 ± 500.93^h^	1125.7 ± 69.4^h^
10% 2-Mercaptoethanol	9578.4 ± 819.73^i^	1327.0 ± 113.57^i^

Data were presented as the mean of three replicates (Mean ± SD). Values followed by the different letters are significantly different at *p* < 0.05

### Determination of K_m_ and V_max_

**Fig. 8:**
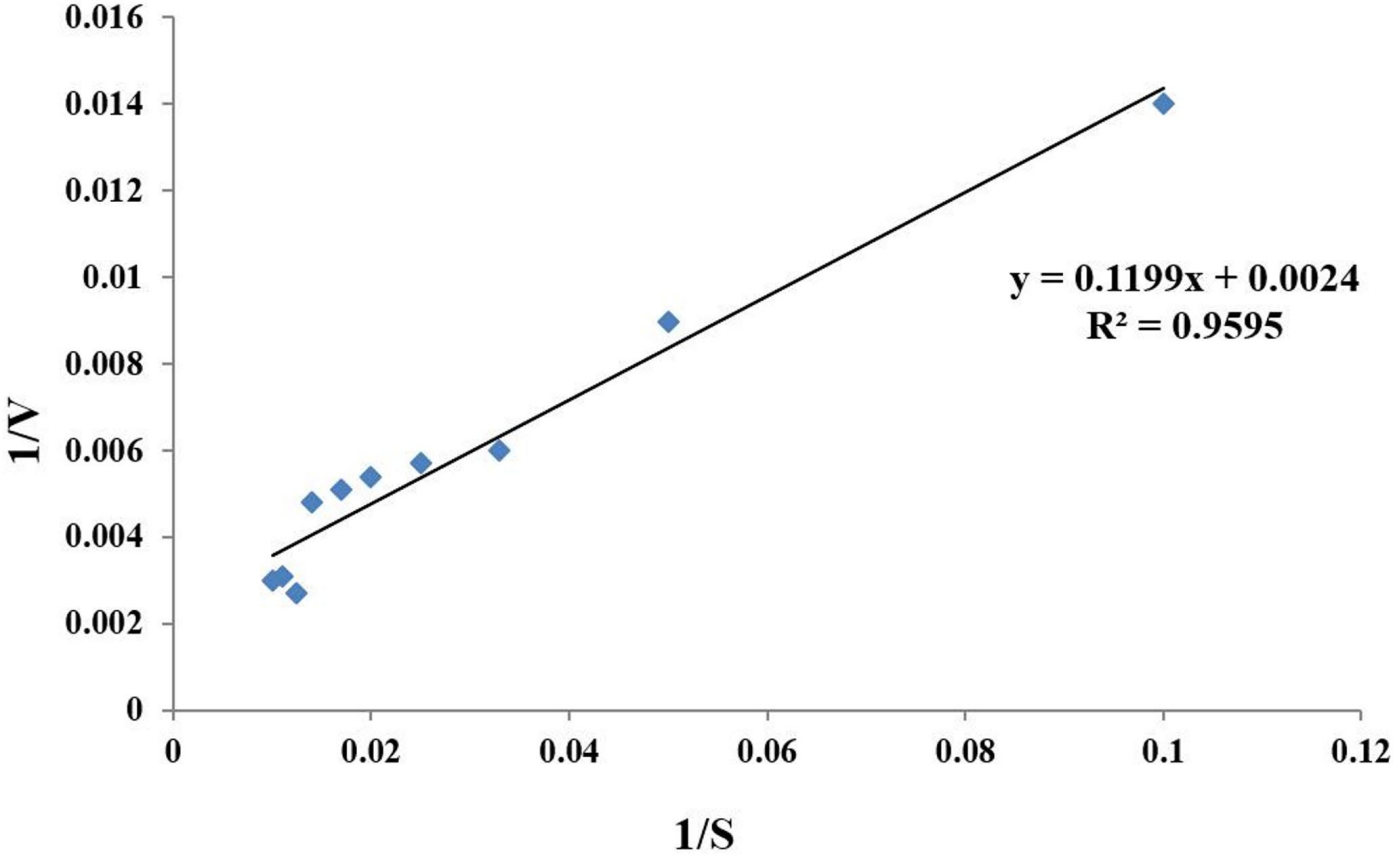
Lineweaver-Burk plot of the reciprocal of initial velocities and keratin concentration

K_m_ and V_max_ were determined from a Lineweaver–Bürk plot to be 49.96 mg/mL and 416.67 µmol/min, respectively (Fig. 8).

### Dehairing activity

**Fig. 9:**
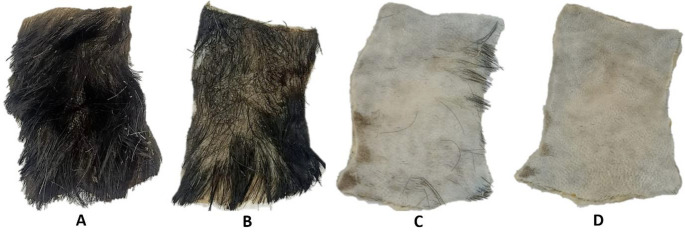
Dehairing activity of the crude cold-active keratinase produced by *P. oxalicum* AUMC 15084 at 20ºC. **(A)** goat skin without treatment, **(B)** goat skin treated for 12 h with the crude keratinase, **(C)** goat skin treated for 16 h, and **(D)** completely dehaired goat skin treated for 20 h

Dehairing potential of *P. oxalicum* AUMC 15084’ crude keratinase using goat skin revealed that complete dehairing was observed after 20 h at 20ºC of treatment with the crude preparation. The goat skin after dehairing and washing with distilled water was soft and stretchable with visible pores indicating the loss of hairs (Fig. 9).

## Discussion

Many biotechnological applications, including the promotion of plant growth, the treatment of keratinous wastes from the leather and agriculture industries, the detergent and textile industries, the decontamination of prions, the treatment of dermatophytes and nail diseases, scars, and epithelial regeneration, have been shown to benefit greatly from the use of microbial keratinases (Vidmar and Vodovnik 2018). Finding efficient keratin degraders opens up possibilities to clean up waste from the cattle, poultry, and leather industries. Research on keratinases aims to break down different waste components (Bhange et al. 2016). Using naturally occurring keratinophilic microorganisms to manufacture enzymes has the advantages of being inexpensive and having no negative effects (Kang et al. 2018; Kalaikumari et al. 2019; de Menezes et al. 2021). Research into the mechanisms behind the breakdown of keratinous materials is still required. According to (Kalaikumari et al. 2019), keratinases can catalyze keratinolysis alone. However, for optimal results, they should be combined with other enzymes like collagenase (Al-Bedak et al. 2023) or disulfide reductases (Kang et al. 2021), which break down disulfide bonds.

In the current investigation, *P. oxalicum* AUMC 15084 was used to hydrolyze the native chicken feathers wastes at low incubation temperatures to produce cold-active keratinase. Even though they grow slowly, some psychrophiles, including *Penicillium* species, have been demonstrated to have high metabolic rates as a physiological response to cold temperatures (Hassan et al. 2016). This makes it easier in making up for insufficient biological reactions at low temperatures. Because they need a lot of ATP to maintain a high metabolic rate, psychrophiles create cold-adapted enzymes that can effectively work at low temperatures, allowing them to make advantage of whatever nutrition sources are accessible in their environment (Al-Maqtari et al. 2019). *Penicillium* species, which are psychrophilic fungi, have been isolated from Antarctic soil probes (Litova et al. 2014). Sea ice, lakes, glaciers, soils, and oceans make up more than 70% of the ecosphere’s cold ecosystems. Antarctica, which is the southernmost continent, is also the coldest due to its 90% cover of ice sheets (Kuddus et al. 2024). Today’s biotechnological industries require macromolecules that are exceptionally robust. Microorganisms adapt to different environments, which leads to the evolution of their molecular machinery. Cold-active enzymes are produced by microorganisms that have acclimated to the cold. Microorganisms that are psychrophilic or psychrotolerant, together with the proteins and enzymes that have evolved to be resistant to cold, offer a plethora of biotechnological applications (Rawat et al. 2024; Sarsan et al. 2024).

The majority of the extracellular enzymes produced by microorganisms are impacted greatly by physicochemical and nutritional variables. Optimizing the various media components in a process based on the generation of biologically active substances by microorganisms can have a substantial impact on production costs and result in either profit or loss. Therefore, it is crucial to look into the ideal circumstances for biotechnology solutions. The appropriate mix of several media components dictates how each component is used. In this investigation, 359.42 U/mL of a cold-active keratinase was synthesized by *P. oxalicum* AUMC 15084 utilizing yeast extract as a nitrogen source, and optimal conditions were set for six days of incubation at pH 8.0 and 15 °C. There are many different types of bacteria and fungi that may generate keratinase, and their optimal fermentation parameters vary greatly. Nevertheless, extremely uncommon information regarding keratinase synthesis that is active at freezing temperatures. *Aspergillus terreus*, for example, produced keratinase at pH 8 and 40 °C for a duration of 25 days (Koutb et al. 2012). Different strains of *Aspergillus niger* produced varying amounts of keratinases; after seven days of incubation with pH 5 producing the highest activity (Mazotto et al. 2013). After four days at pH 6.0 and 30 °C, *Aspergillus* sp. DHE7 had the maximum keratinase activity of 199 U/mL (El-Ghonemy and Ali 2017). After 15 days at pH 9.5 and 30 °C, *Cochliobolus hawaiiensis* produced the greatest amount of alkaline keratinase (Isaac and Abu-Tahon 2016). *Chrysosporium tropicum* generated keratinase at its peak level for 21 days at 25 °C (Menon et al. 2020). *Trichophyton ajelloi* showed maximum enzyme activity at 30 °C (Kačinová et al. 2014). *Microsporum gypseum* and *M. canis* had the maximum activity on the 20th day of incubation (Peng et al. 2020). *Bacillus thuringiensis* showed maximum activity at pH 9.0 and 50 °C (Hassan et al. 2020b). It is often difficult to compare the values of enzyme activity between different investigations due to minor methodological discrepancies. As such, while drawing similarities, one should approach with caution. According to the research, different fungi have extremely varying optimal circumstances. This means that the ideal culture conditions and inhibitory substances must be investigated.

Using ethyl alcohol, an MP 800 ion exchanger, and Sephacryl S 200, the cold-active keratinase of *P. oxalicum* AUMC 15084 was purified for this investigation. A 4.13-fold purification with a specific activity of 684.46 U/mg and a yield of 5.34% was obtained. The optimum pH and temperature for the pure keratinase to exhibit its specific activity peak of 721.8 U/mg were 9.0 and 20 °C, respectively. According to SDS-PAGE, the purified keratinase used in this study has a molecular weight of 37.51 kDa. The keratinase from *Scopulariopsis brevicaulis* was 6.9-fold purified with a 24.1% yield and a maximal specific activity of 70.6 kU/mg using ammonium sulphate, DEAE-Cellulose, and Sephadex G-100. It’s ideal pH and temperature were 8.0 and 40ºC, respectively, and its molecular weight was 39 kDa (Anbu et al. 2005).The keratinase produced by *Aspergillus flavus* K-03 was 11.53 times purified using ammonium sulfate, Sephadex G-100, and the DEAE-Sephadex A-50 ion exchanger, exhibiting a specific activity of 315.16 U/mg and a molecular weight of 31 kDa and an optimum pH and temperature of 8.0 and 45ºC (Kim 2007). Keratinase produced by *A. flavipes* was purified 2.67 times with 4.4% yield using ammonium sulphate, a DEAE-cellulose anion exchanger, and Sephadex G 200 gel filtration, yielding maximum specific activity of 55.5 U/mg at pH 7.0 and a molecular weight of 60 kDa (El-Ayouty et al. 2012). An alkaline condition hastens the breakdown of keratin by participating in the disulfide bond breaking process (Tork et al. 2016), and alkaline keratinases are more appropriate for the leather industry and the production of laundry detergents (Jaouadi et al. 2010; Rai and Mukherjee 2011; Abdel-Naby et al. 2016). Among many other microorganisms, cold-adapted enzymes have been found in *Penicillium*, *Rhodotorula*, and *Pseudomonas*. Research on cold-adapted catalytic processes and cold-adapted enzyme molecular changes has been published on a regular basis (Kumar et al. 2021).

Nevertheless, up to now, studies and publications on the uses of microbes’ cold-adapted enzymes have been scattered (Liu et al. 2023). Any enzyme’s necessary level of purification varies depending on how it is intended to be used; typically, industrial applications like the food industry or detergents demand a low level of purity (Queiroz et al. 2001; Corrêa et al. 2010). In this investigation, addition of various ions has varying impacts on keratinase activity. Mg^2+^, Zn^2+^. Mn^2+^ ions significantly (p < 0.05) raised activity of the pure *P. oxalicum* AUMC 15084’ keratinase. Na^+^, K^+^, Ca^2+^, Co^2+^, Cu^2+^, Ni^2+^, and Fe^2+^ all reduced the keratinase’s activity. Many studies have investigated the impact of metals, activators, and inhibitors on microbial keratinase activity (Alwakeel et al. 2021; Al-Bedak et al. 2023; Li et al. 2024; Ramalingum et al. 2024). This was also true of our strain *P. oxalicum* AUMC 15,084. A feather-degrading culture of *Aspergillus oryzae* has stimulated by Ca^2+^ and Ba^2+^ ions while inhibited by EDTA and Pb^2+^ ions (Farag and Hassan 2004). EDTA, Hg^2+^, and Fe^3+^ significantly decreased the *A. flavipes*’ keratinase activity, while Zn^2+^, Mg^2+^, and Cu^2+^ had no effect on *A. flavipes* keratinase (El-Ayouty et al. 2012). In this investigation, the keratinase activity of *P. oxalicum* AUMC 15084 was greatly increased by the addition of SDS, DMSO, and 2-mercaptoethanol, whereas PMSF inhibited it, indicating that it is a serine protease. The substantial increase in keratinase activity seen in this study can be ascribed to the strong reducing characteristics of SDS, DMSO, and 2-mercaptoethanol, which contribute to the breakdown of disulfide bonds inside keratin molecules. Conversely, several microbial keratinases have been found to be inhibited by SDS (Riffel et al. 2007; Tatineni et al. 2008). In line with our results, PMSF and EDTA completely inhibited the keratinase of *Aspergillus flavus* K-03 (Kim 2007). EDTA and PMSF reduced the keratinase generated by *Bacillus* sp. P7, but SDS enhanced it (Corrêa et al. 2010). PMSF completely suppressed the keratinases generated by *A. ustus* NIOCC #20 (Damare et al. 2006) and *Scopulariopsis brevicaulis* (Anbu et al. 2005). There is an urgent need for more strong and stable keratinases that can be used to lessen the burden of environmental contamination, given the rise in demand for keratinases in the leather, poultry, animal feed, and other industries (Batool et al. 2021).

This study evaluated *P. oxalicum* AUMC 15084’s crude keratinase’s dehairing capacity using goat skin. Twelve hours of treatment with the crude preparation at 20ºC resulted in full dehairing. After dehairing and washing with distilled water, the goat’s skin was elastic and silky. Many studies investigated the dehairing capability of keratinases produced by several fungal and bacterial species. In line with this concern, keratinases produced by *Bacillus* sp. D2 strain (Batool et al. 2021), *Bacillus* sp. MD24 (Suharti et al. 2018), *Trichoderma harzianum* MH-20 (Ismail et al. 2012) were used to enzymatically cure goat and cow hide, resulting in the dehairing and removal of scud and keratin skin layer, leaving the skin surface smooth and undamaged.

## Conclusions

After screening 32 *Penicillium* and *Talaromyces* isolates for keratinolytic activity at 5, 10, and 15 ºC, a *P. oxalicum* strain was found to be most powerful at 10 ºC. After 6 days at pH 8.0 and 15 °C with 0.2% yeast extract as nitrogen, the strain had the maximum keratinase activity. Keratinase was purified 4.13-fold using MP 800 anion exchanger and Sephacryl S 200 h. The SDS-PAGE investigation revealed a 37.51 kD keratinase with optimum activity at pH 9.0 and 20 ºC. Mg^2+^, Zn^2+^, and Mn^2+^ increased keratinase activity by 156.0%, 140.6%, and 156.0 ± 2.9%, respectively. SDS, mercaptoethanol, and DMSO highly increased keratinase activity. *P. oxalicum* AUMC 15084 crude keratinase effectively dehaired goat skin after 20 h at 20 ºC. After dehairing and washing with distilled water, goat skin was smooth and stretchable with visible pores, indicating hair loss.

## Data Availability

No datasets were generated or analysed during the current study.
